# A new perspective on Alzheimer’s disease: microRNAs and circular RNAs

**DOI:** 10.3389/fgene.2023.1231486

**Published:** 2023-09-15

**Authors:** Shahidee Zainal Abidin, Nurul Asykin Mat Pauzi, Nur Izzati Mansor, Nurul Iffah Mohd Isa, Adila A. Hamid

**Affiliations:** ^1^ Faculty of Science and Marine Environment, Universiti Malaysia Terengganu, Terengganu, Malaysia; ^2^ Biological Security and Sustainability (BIOSIS) Research Interest Group, Faculty of Science and Marine Environment, Universiti Malaysia Terengganu, Terengganu, Malaysia; ^3^ Department of Nursing, Faculty of Medicine, Universiti Kebangsaan Malaysia, Kuala Lumpur, Malaysia; ^4^ Department of Physiology, Faculty of Medicine, Universiti Kebangsaan Malaysia, Kuala Lumpur, Malaysia

**Keywords:** microRNA, circular RNA, Alzheimer’s disease, neurodegenerative disease, biomarkers

## Abstract

microRNAs (miRNAs) play a multifaceted role in the pathogenesis of Alzheimer’s disease (AD). miRNAs regulate several aspects of the disease, such as Aβ metabolism, tau phosphorylation, neuroinflammation, and synaptic function. The dynamic interaction between miRNAs and their target genes depends upon various factors, including the subcellular localization of miRNAs, the relative abundance of miRNAs and target mRNAs, and the affinity of miRNA-mRNA interactions. The miRNAs are released into extracellular fluids and subsequently conveyed to specific target cells through various modes of transportation, such as exosomes. In comparison, circular RNAs (circRNAs) are non-coding RNA (ncRNA) characterized by their covalently closed continuous loops. In contrast to linear RNA, RNA molecules are circularized by forming covalent bonds between the 3′and 5′ends. CircRNA regulates gene expression through interaction with miRNAs at either the transcriptional or post-transcriptional level, even though their precise functions and mechanisms of gene regulation remain to be elucidated. The current stage of research on miRNA expression profiles for diagnostic purposes in complex disorders such as Alzheimer’s disease is still in its early phase, primarily due to the intricate nature of the underlying pathological causes, which encompass a diverse range of pathways and targets. Hence, this review comprehensively addressed the alteration of miRNA expression across diverse sources such as peripheral blood, exosome, cerebrospinal fluid, and brain in AD patients. This review also addresses the nascent involvement of circRNAs in the pathogenesis of AD and their prospective utility as biomarkers and therapeutic targets for these conditions in future research.

## 1 Introduction

Alzheimer’s disease (AD) is a common form of dementia that causes progressive mental decline in the elderly. This is due to impaired neurons and synapses, eventually changing patients’ behavior, memory, language, and cognition (Lu et al., 2021). AD is characterized by the accumulation of disposition of amyloid-β (Aβ), neurofibrillary tangle (NFT) formation, and extensive neuronal degeneration (Maciotta et al., 2013). The etiology of AD remains incompletely comprehended. The early indications of AD are frequently characterized by mild symptoms, including memory lapses and challenges in recollecting recent occurrences. As the disease advances, affected individuals may manifest symptoms such as cognitive impairment, perceptual disorientation, linguistic deficits, affective instability, and behavioral alterations.

The utilization of microRNA (miRNA) panels, which consist of multiple miRNAs, has been studied by researchers to enhance diagnostic accuracy. The panels typically consist of a group of differentially expressed miRNAs that, when combined, provide a more comprehensive disease profile. miRNA is a short non-coding RNA that plays an essential role in biological and pathological mechanisms in humans, animals, and plants (Harper et al., 2019; Toivonen et al., 2020; Beylerli et al., 2022). In recent years, many miRNAs have been studied and found to be involved with the development of neurodegenerative diseases, including AD. This makes them a potential biomarker for neurodegenerative diseases.

On the other hand, circular RNAs (CircRNAs) function as regulators of miRNA activity by sequestering them as miRNA sponges or competitive endogenous RNAs (ceRNAs) due to the presence of miRNA binding sites within circRNAs (Hansen et al., 2013a). When miRNAs form complexes with circRNAs, they undergo a process of sequestration or “sponging” from their respective target messenger RNAs (mRNAs) (Hansen et al., 2013b). Furthermore, circRNA regulates host gene expression through various mechanisms, such as acting as protein sponges, facilitating protein translocation and translation, and promoting protein-protein interaction (Zhao et al., 2022). This interaction can be especially important when the targeted miRNAs regulate biological processes or disease pathways. This review provides an overview of miRNAs and circRNAs’ biosynthetic mechanisms and functional roles. Additionally, the most relevant miRNAs and circRNAs, as well as the relationship miRNA-circRNA linked to AD, are discussed. Furthermore, our attention will be directed toward how the levels of multiple miRNAs in peripheral blood, serum, exosomes derived from serum, cerebrospinal fluid, and brain are altered in individuals with AD.

## 2 Biogenesis of microRNA and circular RNA

Most miRNAs are known to be synthesized via a canonical pathway, which undergoes several processing steps before developing into functional miRNAs. In the canonical pathway, primary miRNAs (pri-miRNAs) are transcribed by RNA polymerase II within the nucleus, which generates distinguish hairpin structures with polyadenylated and capped. The hairpin structures within pri-miRNAs are recognized by DGCR8 (DiGeorge syndrome critical region gene 8) and cleaved DROSHA to form precursor miRNAs (pre-miRNAs). The pre-miRNAs are exported to the cytoplasm by exportin 5 (XPO5) via Ran-GTP-dependent mechanisms (Komatsu et al., 2023).

The pre-miRNA is further cleaved off by dicer and its catalytic partner trans-activator RNA-binding protein (TRBP) into ∼22 nt miRNA duplexes. The miRNA duplex is loaded into Argonaute (AGO) protein before unwinding into two strands, guide, and passenger strand. Generally, the RNA-induced silencing complex’s component 3 promoter (C3PO) (RISC) will degrade the passenger strand. At the same time, the guide strand will be incorporated with AGO2 to initiate the RNA silencing mechanism. In some cases, both strands can be incorporated into the RISC complex. The RISC complex then recognizes target mRNAs based on sequence complementary between the miRNA and the mRNA 3′untranslated region (UTR). This recognition leads to mRNA degradation or translation inhibition, subsequently reducing protein expression.

CircRNAs exist as a continuous loop structure. circRNAs are ubiquitously expressed in mammalian cells and can also be found in a tissue-specific manner (Han et al., 2018). In contrast to linear mRNA, circRNA is generated through back-splicing, RNA-binding protein (RBP)-mediated circularisation, and exon skipping driven by lariat or intron-pairing mechanisms from pre-messenger RNA (Sekar and Liang, 2019; Guria et al., 2020). This process results in a circular transcript structure that arises from the fusion of the 3′non-co-linearly splice site with either its upstream 5′or another upstream exon during splicing events (Zhou et al., 2020).

The circRNA molecules that contain exonic or exon-intronic sequences are formed through a process known as back-splicing (Patop et al., 2019). CircRNAs can also be produced using lariat-driven circularisation forming a circular exonic RNA with a large lariat of introns (Guria et al., 2020). In addition, the RBP can bind specific motifs in the flanking introns to promote circularisation (Patop et al., 2019). Several splicing factors, including QK1, ADAR (adenine deaminase acting on RNA), ESRP1, and FUS, have been shown to facilitate circRNA production (Conn et al., 2015; Ivanov et al., 2015). Then circRNAs are transported into the cytoplasm in a size-dependent manner through either ATP-dependent RNA helicase DDX39A (also referred to as nuclear RNA helicase URH49) or spliceosome RNA helicase DDX39B (also known as DEAD box protein UAP56) (Huang et al., 2018).

## 3 microRNA and Alzheimer’s disease

Many studies have demonstrated that dysregulation of miRNAs was associated with the pathogenesis of AD. One of the studies identified a set of miRNAs differentially expressed in the brain, cerebrospinal fluid (CSF), and blood of AD patients compared to healthy controls (Takousis et al., 2019). Although miRNAs are predominantly intracellular, a sizeable fraction of them are migratory and can be identified in extracellular fluids (Zen and Zhang, 2012). These miRNAs are known as circulating miRNAs and can be detected in bodily fluids such as blood, urine, saliva, seminal fluid, breast milk, microvesicles, and exosomes (Weber et al., 2010; Vickers et al., 2011; O’Driscoll, 2015). This review presents a comprehensive summary of miRNA expression in three distinct compartments, blood, CSF, and brain tissues, in relation to AD (Table 1).

**TABLE 1:** The expression of miRNAs in peripheral blood, cerebrospinal fluids (CSF) and brain tissues derived from Alzheimer’s patients.

miRNA	Expression pattern	Experimental approach	Patient demographic	Reference
**Whole Blood**				
*miR-112*, *miR-161*, *let-7d-*3p, *miR-5010-*3p, *miR-151a-*3p	Upregulated	Small RNA-Sequencing validated by RT-qPCR	AD patient	Leidinger et al. (2013)
			• Age: 72.7 ± 10.4	
			• MMSE Score: 18.9 ± 3.4	
*miR-103a-*3p, *miR-107*, *miR-532–*5p, *miR-26b-*5p, *let-7f-*5p	Downregulated		MCI patient	
			• Age: 73.9 ± 6.2	
			• MMSE Score: 25.3 ± 1.4	
*miR-9-*5p, *miR-106a-*5p, *miR-106b-*5p, *miR-107*	Downregulated	RT-qPCR	Late-onset AD (>60 years)	Yilmaz et al. (2016)
**Blood (Platelet)**				
*let-7i-*5p, *miR-125a*-5p, *miR-1233*-5p	Downregulated	miRNA microarray validated by RT-qPCR	Aβ(−) MCI	Lee et al. (2020)
			• Age: 74.2 ± 5.67	
			• MMSE Score: 24.5 ± 3.03	
			Aβ(+) MCI	
			• Age: 74.6 ± 2.48	
			• MMSE Score: 24.0 ± 0.53	
**Serum**				
*miR-98*-5p*, miR-885*-5p*, miR-483-*3p*, miR-342*-3p*, miR-191*-5p*, let-7d*-5p	Downregulated	miRNA sequencing validated by RT-qPCR	Age: 77.83 ± 7.40	Tan et al. (2014)
			MMSE Score: 10.45 ± 2.21	
*miR-210*	Downregulated	RT-qPCR	AD patient (Age: 60—84 years)	Zhu et al. (2015)
			MCI patient (Age: 61—82 years)	
*miR-519*	Upregulated	RT-qPCR	Age: 81.36 ± 13.25	Jia and Liu (2016)
*miR-29, miR-125b, miR-223*	Downregulated		MMSE Scores: 18.3 ± 5.2	
*miR-613*	Upregulated	RT-qPCR	MCI patient (Age: 64.8 ± 7.2)	Li et al. (2016)
			DAT patient (Age: 65.5 ± 6.8)	
*miR-455-*3p, *miR-4668*-5p	Upregulated	Microarray validated by RT-qPCR	AD patient	Kumar et al. (2017)
			• Age: 75.3 ± 10.14	
			• MMSE Score: 22.4 ± 4.6	
			MCI patient	
			• Age: 72.4 ± 7.53	
			• MMSE Score: 25 ± 2.05	
*miR-195*-5p*, miR-146a*-5p, *miR-106b*-3p, *miR-20b*-5p, *miR-497*-5p	Upregulated	Small RNA sequencing validated RT-qPCR	Age: 72.1 ± 8.5	Wu et al. (2017)
*miR-93*-5p*, miR-29c*-3p*, miR-125b*-3p, *miR-19b-*3p	Downregulated		MMSE Scores: 28.9 ± 2.9	
*miR-28-*3p	Upregulated	RT-qPCR	AD patient	Zhao et al. (2020)
			• Age: 70.12 ± 2.09	
			• MMSE Score: 15.48 ± 1.68	
			MCI patient	
			• Age: 69.68 ± 2.11	
			• MMSE Score: 22.67 ± 0.73	
***miR-331-*3p**	Downregulated	RT-qPCR	Age: 72.69 ± 6.64	Liu and Lei (2021)
			MMSE Scores: 16.05 ± 2.69	
*miR-6761-*3p, *miR-6747-*3p, *miR-6875-*3p, *miR-6754-*3p, *miR-6736-*3p, *miR-6762-*3p, *miR-6787-*3p, *miR-208a-*5p, *miR-6740-*3p, *miR-6778-*3p, *miR-6753-*3p, *miR-6716-*3p, *miR-4747-*3p, *miR-3646*, *miR-595*, *miR-4435*	Upregulated	Data were obtained from GEO repository (GSE120584)	Patients are composed of MCI, VaD, and DLB with ages >60 years	Lu et al., 2021, Shigemizu et al., 2019
*miR-125a*-3p, ***miR-22*-3p**, *miR-24-*3p, *miR-6131*, *miR-125b-1-*3p	Downregulated			
*miR-128*	Upregulated	RT-qPCR	AD patient	Zhang et al. (2021)
			• Age: 72.57 ± 8.13	
			• MMSE Score: 16.88 ± 2.04	
**Plasma**				
*let-7d-*5p, *let-7g*-5p*, miR-15b*-5p*, miR-142*-3p*, miR-191-*5p*, miR-545-*3p, *miR-301a-3p*	Downregulated	Nanostring platform validated by RT-qPCR	Cohort 1 (Composed of AD and MCI patients)	Kumar et al. (2013)
			• Age: 78.65 ± 6	
			• MMSE Score: 20.95 ± 4.4	
			Cohort 2 (Only AD patient)	
			• Age: 69.3 ± 6.18	
			• MMSE Score: 16.5 ± 3.97	
*miR-34c*	Upregulated	RT-qPCR	Age: 56–90	Bhatnagar et al. (2014)
			Sporadic late-onset AD	
*miR-34a*, *miR-146a*	Downregulated	RT-qPCR	Age: 80.7 ± 5.8	Kiko et al. (2014)
			MMSE Score: 21.1 ± 3.5	
			CSF-Aβ (1–42) (pg/mL): 378.4 ± 112.2	
			CSF-tau (pg/mL): 480.7 ± 218.9	
			Consist of cerebral atrophy no cerebrovascular lesion	
***miR-384***	Downregulated	RT-qPCR	MCI patient (Age: 63.2 ± 6.1)	Liu et al. (2014)
			DAT patient (Age: 64.2 ± 5.8)	
*miR-483*-5p, *miR-486*-5p, *miR-200a-*3p, *miR-502*-3p	Upregulated	miRCURY LNA miRNA PCR panel	AD patient	Nagaraj et al. (2017)
			• Age: 67 ± 8	
			• MMSE Score: 20.4 ± 4	
			• Tau (pg/mL): 774.3 ± 347	
			• pTau (pg/mL): 94.8 ± 38	
			• Aβ_1-42_ (pg/mL): 387.4 ± 163	
*miR-30b*-5p, *miR-142*-3p	Downregulated		MCI patient	
			• Age: 62 ± 6	
			• MMSE Score: 25.9 ± 2	
			• Tau (pg/mL): 444 ± 243	
			• pTau (pg/mL): 67.3 ± 30	
			• Aβ_1-42_ (pg/mL): 631.9 ± 351	
*miR-1908*	Upregulated	RT-qPCR	Age: 71.3 ± 2.5	Wang et al. (2018)
			MMSE score: 16.8 ± 3.5	
			CDR score: 1.5 ± 0.3	
*miR-103*, *miR-107*	Downregulated	RT-qPCR	Age: 72.5 ± 7.7	Wang et al. (2020)
			MMSE Score: 16.8 ± 3.0	
			Mild dementia	
**Serum (Exosome)**				
*miR-135a,* ***miR-384***	Upregulated	RT-qPCR	MCI (Age: 61.63 ± 7.32)	Yang et al. (2018a)
*miR-193b*	Downregulated		DAT (Age: 74.15 ± 7.93)	
***miR-22-*3p**, *miR-378a-*3p	Upregulated	miRNA sequencing validated by RT-qPCR	Age: 75.1 ± 7.5	Dong et al. (2021)
*miR-30b*-5p	Downregulated			
*miR-342*-5p	Downregulated	RT-qPCR	Age: 75.1 ± 7.5	Dong et al. (2022)
			MMSE score: 6.55 ± 2.86	
**Plasma (Exosome)**				
*miR-342*-3p, *miR-141-*3p, *miR-342*-5p, *miR-23b-*3p, ***miR-125b-*5p**, *miR-24–*3p, *miR-152–*3p	Downregulated	Small RNA deep sequencing	Age: Between 50—75	Lugli et al. (2015)
*miR-423-*5p, *miR-369*-5p, *miR-23a*-5p	Upregulated	Small RNA sequencing	Age: 67.8 ± 5.72	Nie et al. (2020)
*miR-204*-5p*, miR-125a*-5p, *miR-1468*-5p*, miR-375*, *let-7e*-5p	Downregulated		MMSE Score: 13.8 ± 7.43	
**Cerebrospinal fluids**				
*let-7b*	Upregulated	RT-qPCR	Age: 67.84 ± 6.6	Lehmann et al. (2012)
			MMSE Score: 22.54 ± 3.13 t-Tau (pg/mL): 685.77 ± 282.23	
			Aβ_1-42_ (pg/mL): 408.38 ± 89.34	
*miR-27a-*3p	Downregulated	RT-qPCR	Cohort 1	Frigerio et al. (2013)
			• Age: 66.6 ± 6.9	
			• MMSE Score: 24.4 ± 2.2	
			• Tau (pg/mL): 677 ± 309.6	
			• pTau (pg/mL): 93.6 ± 36.3	
			• Aβ_1-42_ (pg/mL): 334 ± 131.8	
			Cohort 2	
			• Age: 75.8 ± 8.6	
			• Tau (pg/mL): 864.7 ± 298	
			• pTau (pg/mL): 122.1 ± 26	
			• Aβ_1-42_ (pg/mL): 812.2 ± 213.5	
*miR-29a*, *miR-29b*	Upregulated	RT-qPCR	Age: 80.7 ± 5.8	Kiko et al. (2014)
			MMSE Score: 21.1 ± 3.5	
			CSF-Aβ (1–42) (pg/mL): 378.4 ± 112.2	
*miR-34a*, *miR-125b*, ***miR-146a***	Downregulated		CSF-tau (pg/mL): 480.7 ± 218.9	
			Consist of cerebral atrophy	
***miR-384***	Downregulated	RT-qPCR	MCI patient (Age: 63.2 ± 6.1)	Liu et al. (2014)
			DAT patient (Age: 64.2 ± 5.8)	
***miR-146a*** *, miR-100, miR-505#, miR-4467, miR-766, miR-3622b*-3p, *miR-296*	Upregulated	TaqMan OpenArray Human MicroRNA	Age: 72.1 ± 8.5	Denk et al. (2015)
*miR-1274a*, *miR-375, miR-708, , miR-219, miR-103*	Downregulated		Total tau (pg/mL): 708.5 ± 282.9 pTau (pg/mL): 92 ± 93.3 Aβ1-42 (pg/mL): 446.7 ± 164.1	
*miR-210*	Downregulated	RT-qPCR	AD patient (Age: 60—84 years)	Zhu et al. (2015)
			MCI patient (Age: 61—82 years)	
			MCI patient (Age: 64.8 ± 7.2)	
			DAT patient (Age: 65.5 ± 6.8)	
*miR-613*	Upregulated	RT-qPCR		Li et al. (2016)
*miR-29a*	Upregulated	RT-qPCR	Age: 70.4 ± 9.1	Müller et al. (2016)
			MMSE Score: 19.7 ± 3.2	
*miR-378a-*3p*, *miR-1291*	Upregulated	TaqMan microRNA array	Age: 69.62 ± 7.27	Lusardi et al. (2017)
*miR-143*-3p, *miR-142*-3p, *miR-328*-3p, *miR-193a*-5p, *miR-19b*-3p, *miR-30d-*5p, *miR-340*-5p, *miR-140*-5p, ***miR-125b*-5p**, *miR-223*-3p	Downregulated		MMSE Score: 18.28 ± 6.40	
*let-7b*, *let-7e*	Upregulated	RT-qPCR	AD patient	Derkow et al. (2018)
			• Age: 71.5 ± 8.5	
			• MMSE Score: 21.9 ± 4.6	
			• Tau (pg/mL): 615.6 ± 322.7	
			• Aβ_1-42_ (pg/mL): 523.8 ± 94.9	
			FTD patient	
			• Age: 64 ± 11.5	
			• MMSE Score: 22.9 ± 4.9	
			• Tau (pg/mL): 430.6 ± 195.5	
			• Aβ_1-42_ (pg/mL): 1,227 ± 524.4	
***miR-125*-5p**	Upregulated	miRNA array validated by RT-qPCR	YOAD patient	McKeever et al. (2018)
			• Age: 60.9 ± 4.6	
			• Memory immediate recall: 8.9 ± 4.6	
			• Delayed recall: 2.8 ± 4.4	
			• Delayed recognition: 15.3 ± 3.7	
			• Visuospatial: 15.7 ± 10.7	
			• Aβ_1-42_ (pg/mL): 356.0 ± 159.1	
*miR-451a*, *miR-605*-5p	Downregulated		• Total tau (pg/mL): 744.5 ± 375.0	
			• pTau (pg/mL): 101.7 ± 37.9	
			LOAD patient	
			• Age: 75.5 ± 4.6	
			• Aβ_1-42_ (pg/mL): 431.3 ± 139.4	
			• Tau (pg/mL): 721.6 ± 245.1	
			• pTau (pg/mL): 97.1 ± 19.7	
*miR-455*-3p	Upregulated	RT-qPCR	Age: 79.09 ± 6.17	Kumar and Reddy (2021b)
*miR-16*-5p, ***miR-331***-3p*, miR-409*-3p, *miR-454-3p*	Upregulated	miRNA array validated by RT-qPCR	AD e−3,3	Sandau et al. (2022)
			• Age: 72.9 ± 10.0	
			• MMSE score: 21.8 ± 3.6	
			AD e−3,4	
			• Age: 73.3 ± 5.1	
			• MMSE score: 21.2 ± 2.2	
**Brain (Hippocampus)**				
*miR-140*	Upregulated	RT-qPCR	• Age: 82 ± 7	Akhter et al. (2018)
			• Plague score: Sparse—Frequent	
			Braak stage: IV-VI	
*miR-142-*5p, *miR-146-*5p, *miR-155-*5p, *miR-455-*5p	Upregulated	RT-qPCR	Hippocampal tissue samples were obtained from the London Neurodegenerative Diseases Brain Bank	Sierksma et al. (2018)
*miR-143-*3p	Downregulated	RT-qPCR	Age: 79.33 ± 12.80	Wang et al. (2022b)
			CERAD: C	
**Brain (Entorhinal Cortex)**				
*miR-101*-3p	Upregulated	RT-qPCR	Braak stage I - II	Kikuchi et al. (2020)
			• Age: 78.5 ± 2.4	
			Braak stage III - IV	
			• Age: 82.6 ± 4.7	
			Braak stage V - VI	
			• Age: 82.9 ± 4.7	
**Brain (Frontal Cortex)**				
*miR-212, miR-23a*	Downregulated	miRNA microarray validated by RT-qPCR	AD patient	Weinberg et al. (2015)
			• Age: 88.6 ± 7.0	
			• MMSE Score: 17.5 ± 8.1	
			• Global cognitive score: −1.3 ± 0.8	
			MCI patient	
			• Age: 82.9 ± 4.9	
			• MMSE Score: 28.0 ± 1.3	
			• Global cognitive score: −0.03 ± 0.4	
*miR-346*	Downregulated	RT-qPCR	Age: 80.8 ± 1.7	Long et al. (2019)
**Brain (Broadman**’**s Area 10)**				
*miR-455*-3p	Upregulated	RT-qPCR	Age: 79.85 ± 8.29	Kumar and Reddy (2018)

Data were shown as Mean ± SD; MMSE, Mini-Mental State Examination; MCI, mild cognitive impairment; VaD, vascular dementia; DLB, dementia with lewy bodies; CDR, clinical dementia rating; DAT, dementia of the alzheimer type; FTD, frontotemporal disorder; YOAD, Young On-Set Alzheimer’s Disease; LOAD, Late On-Set Alzheimer’s Disease; CERAD, The Consortium to Establish a Registry for Alzheimer’s Disease. Contradictory results were seen for the miRNA (bold).

### 3.1 Blood sample

miRNAs exhibit considerable stability in blood and can be conveniently obtained through minimally invasive procedures. Studies have revealed distinct miRNAs with altered expression profiles in the bloodstreams of AD patients compared to those in good health. The miRNAs that exhibit differential expression have the potential to be used as biomarkers in the diagnosis and monitoring of progression in AD. Previous studies have shown differences in the expression profile of miRNAs between various blood components, including whole blood, serum, plasma, and platelets (Table 1). However, only a few miRNAs demonstrate similar expression patterns. Out of the miRNAs that were assessed for their expression patterns, only miR-107 and miR-125a-5p were found to have similar expression profiles in both whole blood and plasma (Leidinger et al., 2013; Yilmaz et al., 2016; Lee et al., 2020; Nie et al., 2020; Wang et al., 2020; Lu et al., 2021). In contrast, there were seven miRNAs (let-7d-5p, miR-384, miR-191-5p, miR-24-3p, miR-30b-5p, miR-342-3p, and miR-342-5p) that showed similar expression profiles between serum and plasma. (Kumar et al., 2013; Liu et al., 2014; Tan et al., 2014; Lugli et al., 2015; Nagaraj et al., 2017; Yang et al., 2018; Dong et al., 2022; 2021; Lu et al., 2021).

The miR-107 was found to be downregulated in plasma, blood, and platelet. This miRNA was found to be involved in the initiation of blood coagulation. In addition, miR-107, together with other miRNAs such as miR-96, miR-200b, miR-485, miR-107, and miR-223, play a crucial role in platelet, specifically in reactivity, aggregation, secretion, and adhesion (Gatsiou et al., 2012). A previous study has indicated that the levels miR-107 were reduced in the neocortex of individuals with AD compared to the control group (Nelson and Wang, 2010). Furthermore, it has been discovered that miR-107 has targeted 3′UTR of β-site amyloid protein-cleaving enzyme 1 (BACE1) that participates in Aβ production (Wang et al., 2008). In the progression of AD, there was a tendency for the levels of BACE1 mRNA to rise concomitantly with the decline in miR-107 levels.

Besides miR-107, miR-125a-5p has been implicated in regulating von Willebrand factor (VWF), a protein involved in platelet adhesion and aggregation (Bhatlekar et al., 2020). In a study of patients with acute myocardial infarction, decreased levels of miR-125a-5p were associated with increased VWF expression and platelet activation, suggesting that miR-125a-5p may have a role in regulating platelet function and blood clotting. While this study did not specifically investigate the role of miR-125a-5p in AD, the involvement of this miRNA in regulating blood clotting factors suggests they may play a role in AD pathogenesis. Growing evidence indicates that blood clotting factors play a role in the development and progression of AD (Paul et al., 2007; Zamolodchikov et al., 2015). One of the most widely studied clotting factors in relation to AD is fibrinogen, which is involved in blood clotting and inflammation.

In AD, fibrinogen is bound with Aβ in the brain tissue and blood vessel, thus leading to the fibrillization of Aβ and the formation of fibrin clots resistant to breaking down. Reducing fibrinogen levels lowers cerebral amyloid angiopathy (CAA) and blood-brain barrier (BBB) permeability, reduces microglial activation, and improves cognitive performance in AD mouse models (Cortes-Canteli et al., 2012). Also, a prothrombotic state in AD is shown by more clots forming, less fibrinolysis, and higher amounts of coagulation factors and activated platelets. Abnormal fibrin deposition and persistence in AD may be caused by alterations in blood clotting and Aβ-fibrinogen binding. This could lead to Aβ deposition, reduced cerebral blood flow, worsened neuroinflammation, and eventually neurodegeneration (Cortes-Canteli et al., 2012).

Furthermore, the expression of let-7d-5p, miR-384, miR-191-5p, miR-24-3p, miR-30b-5p, miR-342-3p, and miR-342-5p were downregulated in serum and plasma. The expression of miR-191-5p was reduced in microglia and hippocampal tissues of APP/PS1 mice stimulated with Aβ1-42 (Wan et al., 2021). In addition, the overexpression of miR-191-5p was observed to enhance cell viability and suppress the apoptosis rate in microglia treated with Aβ1-42. The inhibition of Aβ1-42-induced microglial cell injury was due to the inactivation of the MAPK signaling pathway, in which Map3k12 was targeted by miR-191-5p. Moreover, miR-191–5p reduced tau phosphorylation and enhanced neurite outgrowth *in vitro* (Wang et al., 2022a). This study also found that miR-191-5p reduced the levels of phosphorylated amyloid precursor protein (APP) and the generation of Aβ. The negative effects of miR-191-5p on tau phosphorylation, Aβ secretion, and neuronal cell death were due to its direct targeting of DAPK1.

Moreover, the miR-22-3p was also found to have different expressions pattern between serum and serum exosomes (Dong et al., 2021; Lu et al., 2021). Exosomes can be considered a stable source of miRNA since they can prevent RNase degradation and recover the miRNAs (Koga et al., 2011; Thind and Wilson, 2016). This condition can be one of the reasons why the expression pattern was different between serum and serum exosome.

### 3.2 Cerebrospinal fluid (CSF) sample

Besides blood, miRNA profiling also has been performed in AD’s CSF. CSF encompasses the central nervous system (CNS) and is a reliable indicator of the biochemical alterations in this region. Numerous studies have examined miRNA expression in the CSF of individuals with AD, and certain miRNAs exhibit differential expression in AD compared to those deemed healthy controls. Among these miRNAs, only let-7b, miR-125b, miR-146a, and miR-29a were reported in several studies that have deregulation in Alzheimer’s patients (Lehmann et al., 2012; Kiko et al., 2014; Denk et al., 2015; Müller et al., 2016; Lusardi et al., 2017; Derkow et al., 2018; McKeever et al., 2018). However, two studies revealed downregulated miR-125b (Kiko et al., 2014; Lusardi et al., 2017), whereas McKeever et al. found this miRNA increased in AD (McKeever et al., 2018). Additionally, Kiko et al. found that miR-146a was downregulated (Kiko et al., 2014), but Denk et al. found that miR-146a was increased in AD (Denk et al., 2015). The variability in outcomes across studies can be attributed to various unidentified factors.

The study revealed a significant rise in let-7b levels correlated with AD progression. The escalation of let-7b was predominantly attributed to CD4^+^ T cells in the CSF. Nevertheless, the precise role of let-7b in the onset and course of AD has yet to be entirely understood because the involvement of this miRNA in AD is still in the preliminary phases. Based on Pearson correlation coefficients (R) analysis, it was revealed that there was a significant association between the levels of let-7b in the CSF and total tau (*t*-Tau) in the subjective memory complaints (SMC), mild cognitive impairment (MCI), and AD subjects. A significant correlation was also observed between the levels of let-7b and phosphorylated tau (p-Tau) in both MCI and AD subjects. Hence, it can be inferred that let-7b correlates with *t*-Tau and p-Tau, namely, in individuals diagnosed with MCI and AD (Liu et al., 2018).

The overexpression of miR-125b suppressed cellular proliferation, induced apoptotic responses, and enhanced inflammatory and oxidative stress in mouse neuroblastoma Neuro2a APPswe/Δ9 cells. In addition, the upregulation of miR-125b resulted in a significant increase in the expression of APP and β-secretase 1 (BACE1) and the production of Aβ peptide (Jin et al., 2018). Furthermore, overexpression of miR-125b in primary neurons results in the hyperphosphorylation of tau and an increase in the expression of p35, cdk5, and p44/42-MAPK signaling. This miRNA was also found to directly target and downregulate the phosphatases DUSP6 and PPP1CA, subsequently leading to an increase in tau hyperphosphorylation (Banzhaf-Strathmann et al., 2014). On the other hand, another study has reported conflicting results. It was found that the decrease in levels of miR-34a-5p and miR-125b-5p led to an increase in the expression of BACE1 in AD patients and cell cultures (MCN and N2a cells) that were treated with Aβ (Li et al., 2020).

### 3.3 Brain samples

Although obtaining brain tissue samples for miRNA analysis is challenging due to the invasive nature of the procedure, some studies have investigated miRNA expression in post-mortem brain tissue from AD patients. These studies have revealed altered expression of specific miRNAs in different brain regions affected by AD pathology, including but not limited to the hippocampus and frontal cortex. It is important to note that miRNA expression profiles may vary depending on the stage and severity of AD and the brain regions examined. Additionally, the findings from different studies can sometimes be inconsistent, highlighting the need for further study to validate and establish robust miRNA biomarkers for AD.

In AD patients’ brains, five miRNAs (miR-140, miR-142-5p, miR-146-5p, miR-155-5p, and miR-455-5p) were upregulated, and only miR-143-3p was downregulated in the hippocampus (Akhter et al., 2018; Sierksma et al., 2018; Wang et al., 2022b). Other studies demonstrated that miR-101-3p and miR-455-3p were significantly higher in the entorhinal cortex and Broadman’s area 10 of Alzheimer’s patients (Kumar and Reddy, 2018; Kikuchi et al., 2020). While miR-346 was reduced in the frontal cortex of AD (Long et al., 2019).

In APP/PS1 AD mouse model, the expression of miR-455–3p was upregulated, and its targeted gene, cytoplasmic polyadenylation element-binding 1 (CPED1), was downregulated in the hippocampus of the mouse at the age of 9 months (Xiao et al., 2021). Inhibition of CPED1 by miR-455-5p caused suppression of α-Amino-3-hydroxy-5-methyl-4-isoxazole propionic acid (AMPA) receptor expressions, subsequently mediated cognitive deficits. Nevertheless, Kumar et al.’s study showed conflicting results with these findings. Kumar and colleagues have generated a transgenic miR-455-5p (miR-455-3p TG) mouse model and demonstrated that the lifespan of this mouse was 5 months longer than the wild-type mice (Kumar et al., 2021a). However, the knockout (KO) mice had a lifespan that was 4 months shorter than the WT mice. Based on behavior studies, miR-455-3p TG mice exhibited enhanced cognitive behavior, spatial learning, and memory compared to age-matched WT mice and miR-455-3p KO mice.

## 4 Circular RNA and microRNA in Alzheimer’s disease

A new class of non-coding RNAs known as circRNAs has been identified. The circRNAs are produced by a non-canonical splicing event called back-splicing (Kristensen et al., 2019). This RNA was initially identified in 1976 as a viroid consisting of a single-stranded circular RNA molecule isolated from an infected tomato plant (Sanger et al., 1976). This RNA molecule exhibits a structure that is covalently closed-like structure, with a high degree of self-complementarity and base-pairing. The circRNAs have been identified as miRNA sponges due to having highly abundant miRNA binding sites (Hansen et al., 2013b). CircRNAs play a crucial role in developing and maintaining brain homeostasis in the brain. Several studies have shown that a healthy mammalian brain has the highest circRNA expression level and varies across different brain regions (Rybak-Wolf et al., 2015; You et al., 2015; Li et al., 2017).

The circRNAs were highly abundant in the cerebellum, followed by the prefrontal cortex and hippocampus (Rybak-Wolf et al., 2015). Interestingly, circRNAs expression was higher in neurons than in astrocytes in the cerebral cortex and cerebellum (Gokool et al., 2020). In neurons, they are highly concentrated in the synapses of the hippocampus, indicating their potential involvement in synaptic plasticity and cognitive processes (You et al., 2015). Many circRNAs have been shown to interact with disease-associated miRNAs, suggesting that circRNAs could play major roles in the development of diseases and as a prospective prognostic biological marker. For instance, a study by Zhao and co-workers showed that ubiquitin-protein ligase A (UBE2A) expression in AD is affected by the sponging activity of CDR1-as and miR-7 (Zhao et al., 2016). The miR-138-circHDAC9 complex is an additional mechanism that regulates the metabolism of Aβ in AD. The level of circHDAC9 was reduced in the serum of individuals with AD, which may lead to upregulation of miR-138 expression and downregulation of Sirt1 and ADAM10. Consequently, the processing of amyloid APP was redirected from the β-secretase pathway to the β-secretase pathway, resulting in an elevation of amyloid accumulation (Lu et al., 2019).

ciRS-7, the most characterized circRNA, comprises more than 70 conserved binding sites for miR-7 (Hansen et al., 2013b). The ciRS-7 is a robust and stable expression in various tissue, particularly in neural tissues (Ma et al., 2020). The downregulation of ciRS-7 has negatively correlated with miR-7 (Kristensen et al., 2019). The upregulation of miR-7 results in the downregulation of targets associated with AD, namely, UBE2A, which hinders the degradation of APP and β-secretase in the brain (Zhao et al., 2016). Frontotemporal lobar degeneration (FTLD-TAU) patients with 53 MAPT gene mutations are strongly associated with AD (Ghetti et al., 2015). Back-splicing exon 12 to 7 (tau circ 12→7) or 12 to 10 (tau circ 12 → 10), the MAPT gene produces two circRNAs in the brain, contributing to AD pathology. Only tau circ 12 → 7 has a start codon, but 12 → 10 circRNA may require ADAR activity to start translation (Welden et al., 2022). Despite the absence of stop codons in both circRNAs, protein translation can still occur at various locations in circular patterns, producing abnormal proteins. These circRNAs proteins tend to self-aggregate and form neurofibrillary tangles, possibly contributing to FTLD progression (Welden et al., 2022).

Another circRNA involved in tau phosphorylation is circPCCA. The circPCCA might competitively bind to miR-138-5p, inhibiting miR-138-5p′s ability to induce glycogen synthase kinase-3β activation and facilitate tau phosphorylation. These findings indicate that high circPCCA expression can potentially reduce disease severity in AD (Wang et al., 2015; Li et al., 2020).

Based on microarray analysis of CSF derived from AD patients, there was an upregulation of 112 circRNAs and a downregulation of 51 circRNAs when compared to the control subjects (Li et al., 2020). These circRNAs were enriched in pathways associated with AD, such as the neurotrophin signaling pathway, natural killer cell-mediated cytotoxicity, and cholinergic synapse. Further validation using RT-qPCR has demonstrated an increase in the expression of circLPAR1, circAXL, and circGPHN, while circPCCA, circHAUS4, circKIF18B, and circTTC39C were found to be decreased in AD patients.

## 5 Remarks, challenges, and future direction

Despite the intense research on miRNAs expression profiles for diagnosing AD, they are still in their early stage of development. One of the reasons is the complexity of the disorders to understand their pathological cause, which involves many different pathways and targets. Certain miRNAs displayed a distinct expression pattern specific to particular tissues or developmental stages. These miRNAs play a significant role in preserving tissue identity and function by contributing to various biological processes. Several miRNAs have been identified as brain-specific miRNAs, including miR-143, miR-125a/b, miR-138, miR-708, and miR-9 (Guo et al., 2014). Interestingly, these miRNAs have been identified in various other biological compartments, including blood, serum, plasma, and CSF (Table 1), which suggests that these miRNAs have the potential to be valuable candidates for biomarkers in AD in the future.

**FIGURE 1:**
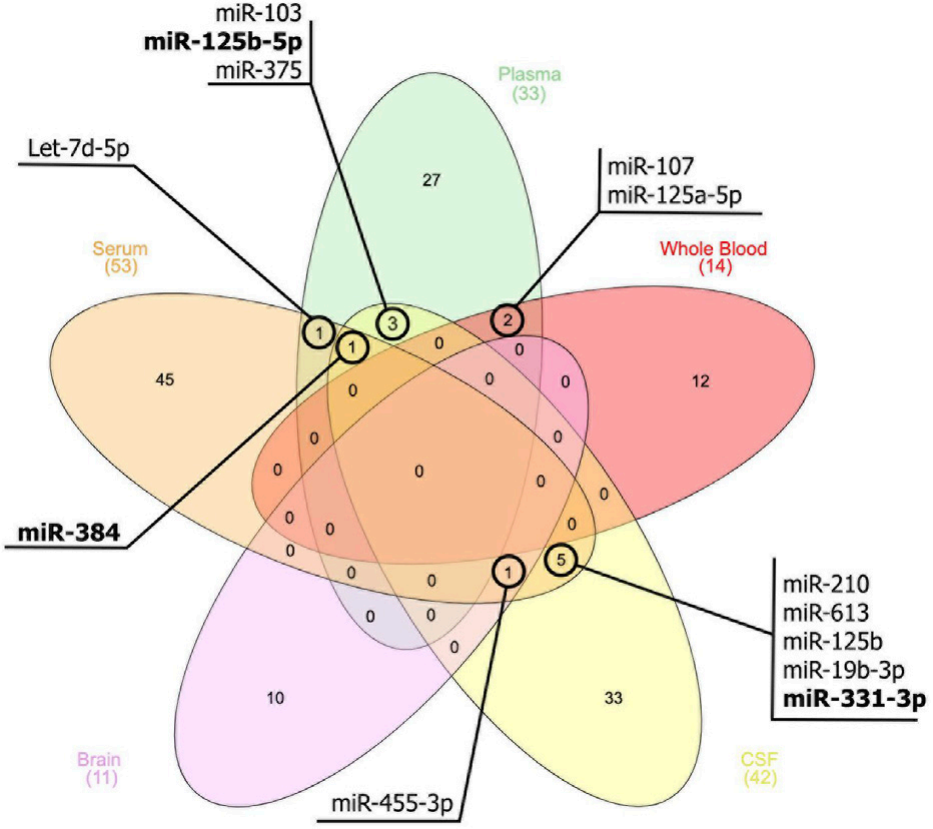
Venn diagram illustrates the miRNAs that exhibit similar expression patterns across peripheral blood, cerebrospinal fluids (CSF), and brain tissue. The miRNAs that are bold indicate a different expression pattern between the samples.

Moreover, based on the thorough assessment of the miRNAs expression profile (Figure 1), this present study identified several miRNAs also hold promise as a potential tool for AD diagnosis, monitoring disease progression, and gaining insights into the underlying molecular mechanisms of the disease. These miRNAs (Figure 1) were found to be associated with genes such as MAPK, BACE1, PTGS2, STAT3, SNAP25, and BDNF. This association subsequently contributes to the development of AD. For instance, the upregulation of miR-125 in AD promotes Tau hyperphosphorylation by regulating MAPK kinases (Banzhaf-Strathmann et al., 2014). This activation is most likely achieved by down-regulating the expression of phosphatase genes that miR-125 targets, which are DUSP6, Bcl-W, and PPP1CA (Banzhaf-Strathmann et al., 2014). BDNF, an additional gene implicated in AD, is linked to cognitive decline, particularly in immediate memory. The expression of miR-613 was found to have a negative regulatory effect on the expression of BDNF (Li et al., 2016). This relationship was observed in the hippocampus of APP/PS1 mice, where high levels of miR-613 coincided with low levels of BDNF expression (Li et al., 2016). In addition, the upregulation of miR-210-5p was observed to cause a reduction in the number of synapses in primary hippocampal neurons (Ren et al., 2018). Conversely, inhibition of miR-210-5p led to an increase in synaptic formation. This condition can be attributed to the downregulation of SNAP25, which is negatively inhibited by miR-210-5p (Ren et al., 2018).

There was a positive correlation between the expression of serum miR-331-3p and MMSE scores in patients with AD (Liu and Lei, 2021). The SH-SY5Y cell viability was significantly increased by overexpressing miR-331-3p while inhibiting miR-331-3p reduced cell viability. Meanwhile, the levels of proinflammatory cytokines, such as IL-1β, IL-6, and TNF-α, were decreased when miR-331-3p was overexpressed. Conversely, when miR-331-3p was knocked down, these cytokine levels increased. The treatment of Aβ resulted in a significant decrease in the expression of miR-331-3p. This finding aligns with the observed reduction of miR-331-3p in serum samples from individuals with AD (Liu and Lei, 2021).

STAT3 phosphorylation was markedly elevated in SH-SY5Y cells after transfection with an inhibitor of miR-29c-3p or miR-19b-3p (Wu et al., 2017). Based on computational predictions, miR-29c-3p, and miR-19b-3p target the same sequence in the 3′-UTR of STAT3. The hippocampus of the AD mice model and AD post-mortem brain shows substantial increases in phosphorylation of STAT3. In addition, STAT3 has served as a transcriptional regulator of BACE1, the crucial enzyme involved in producing Aβ (Millot et al., 2020). Other miRNAs that were observed to decrease their expression when the BACE1 level was increased were miR-16-5p and miR-19b-3p (Wu et al., 207). The overexpression of miR-16-5p or miR-19b-3p reduced the adverse effects of Aβ on cell viability and apoptosis in SH-SY5Y cells. Conversely, the knockdown of these miRNAs promoted the injury induced by Aβ.

In addition to miR-16-5p and miR-19b-3p, miR-107 and miR-384 also have complementary binding sites on the 3′UTR of BACE1, which leads to the suppression of its expression (Wang et al., 2008; Liu et al., 2014; Zhang et al., 2020). The relationship between miR-107 and circ_0049472 was confirmed through a dual-luciferase reporter and RNA pull-down assays. Circ_0049472 was found to be overexpressed in CSF and serum samples derived from AD patients. This gene was also overexpressed in SK-N-SH and CHP-212 cells induced with Aβ. The cell viability and apoptosis induced by the downregulation of circ_0049472 in Aβ-induced SK-N-SH and CHP-212 cells were abolished when miR-107 was silenced (Zheng et al., 2022).

Another gene that is related to AD is APP. A previous study discovered that miR-455 regulates APP by binding to its 3′UTR. This regulation was protective against mitochondrial and synaptic abnormalities induced by mutant APP in AD (Kumar et al., 2019). The PTGS2 was predicted to be targeted by miR-103, and it was discovered that PTGS2 is regulated in the opposite direction by miR-103 expression. In the PC12 cellular AD model, transfection with PTGS2 and miR-103 mimic plasmid decreased total neurite growth compared to the miR-103 mimic group. Additionally, there was an increase in cell apoptosis, suggesting that PTGS2 mimic mitigated the impact of miR-103 mimic on AD progression (Yang et al., 2018). In addition, circ_0000950 decreased the expression of miR-103 and increased the expression of PTGS2 in rat pheochromocytoma cell line PC12 cells and rat cerebral cortex neurons AD models. Overexpression of circ_0000950 resulted in increased neuron apoptosis, decreased neurite outgrowth, and elevated levels of IL-1β, IL-6, and TNF-α. On the other hand, the knockdown of circ_0000950 inhibited neuron apoptosis, promoted neurite outgrowth, and reduced levels of IL-1β, IL-6, and TNF-α. These effects were observed through the direct sponging of miR-103 (Yang et al., 2019).

Certain miRNAs, such as miR-455-3p and miR-210, have been found to possess protective mechanisms. According to Table 1, the expression of these miRNAs was upregulated and downregulated in AD patients, respectively. This condition has raised speculation that these miRNAs are protective in reducing AD severity. There are variations in the expression patterns of different miRNAs, such as miR-331-3p, miR-125-5p, and miR-384, across various biological sources. These changes in their environment potentially lead to their ability to respond, or they may also have a protective role in AD severity. Nevertheless, the precise mechanisms governing the expression of these microRNAs remain unidentified based on current knowledge.

circRNAs are known for their high conservation and structural stability. They have been found to play a crucial role in the onset and progression of numerous diseases. Thus, they could be valuable biomarkers or targets for therapy. Nevertheless, there are still barriers that must be addressed. This includes investigating how circRNAs interact with specific miRNAs, mRNAs, and proteins. By understanding these interactions, we can uncover a regulatory network that plays a role in AD development. To confirm the clinical relevance of circRNAs in AD, further investigation is required to understand their mechanisms and establish correlations. This will involve analyzing a large cohort of patient samples. Furthermore, there is an urgent need for technical advancements in accurately quantifying a specific circRNA and effectively silencing it without impacting the expression of the parent linear transcript.

Also, miRNAs can be detected in the bloodstream and potentially be used as biomarkers for the disease. It is essential to mention that miRNA can be detected in CSF. However, miRNA has the unique ability to traverse the blood-brain barrier and maintain its integrity, protected against degradation. This is achieved by its interaction with protein complexes and its containment within membrane-bound vesicles, such as exosomes (Hill, 2019). miRNA levels in circulation have the potential to indicate neuronal function and dysfunction accurately. This suggests they could be used as innovative therapeutic targets for treating dementia. Several recent meta-analyses have been conducted to establish a unified miRNA signature for AD. For example, in their study, Swarbrick et al. (2019) discovered a peripheral blood microRNA signature comprising ten molecules that could be linked to Braak Stage III.

In contrast, Bottero and Potashkin (2019) made predictions regarding miRNAs that were expected to influence the expression of genes known to exhibit differential expression in individuals with MCI and AD. Diagnostic tools and the significant heterogeneity among studies have limited the use of miRNAs as biomarkers and identifying specific miRNAs associated with AD. The heterogeneity observed in this context can be attributed to several factors, including variations in sample handling and the utilization of different profiling techniques such as microarrays, NGS, RT-qPCR, and other analytical approaches. Hence, further research is required to develop consistent protocols, determine dependable biomarkers, and comprehend the functional consequences of miRNA dysregulation in AD.

## 6 Conclusion

In summary, miRNA and circRNA represent promising avenues for developing non-invasive biomarkers for Alzheimer’s disease. The continued exploration of these non-coding RNA molecules in the context of AD has the potential to revolutionize AD diagnosis, monitoring, and therapeutic interventions, ultimately contributing to better disease management and improved quality of life for affected individuals.
